# Dementia in Germany: epidemiology, trends and challenges

**DOI:** 10.25646/11667

**Published:** 2023-09-20

**Authors:** Daniela Georges, Elena Rakusa, Anna-Victoria Holtz, Anne Fink, Gabriele Doblhammer

**Affiliations:** 1 University Rostock, Germany; 2 German Center for Neurodegenerative Diseases, Bonn, Germany

**Keywords:** POPULATION AGEING, HEALTH TRENDS, DEMENTIA, NEED FOR CARE, DEMENTIA PREVENTION, BURDEN OF DISEASE

## Abstract

**Background:**

Dementia poses a growing challenge for individuals, healthcare, social support, and society amidst the ongoing ageing of populations. To evaluate the care requirements and social implications of dementia in Germany, reliable statistics regarding its current and future occurrence are necessary.

**Methods:**

Using existing data sources and recent research results, this paper compiles and analyses relevant statistics on the occurrence of dementia in Germany, presents protective and risk factors, and options for care provision.

**Results:**

Recent projections indicate a potential surge in the number of dementia patients in Germany, predicted to rise from 1.7 million at present to up to 3.0 million by the year 2070. Cognitive and motor deterioration and behavioural changes associated with dementia lower the ability to live independently. These changes are often tied to social exclusion and stigma and, particularly in the severe phase of the disease, necessitate extensive medical and care requirements. This contributes to dementia being one of the most costly diseases at old age from an overall societal perspective. Currently, there are no curative treatment options available.

**Conclusions:**

To reduce the increase in the number of dementia patients and associated costs in the future, preventive approaches, particularly promoting a healthy lifestyle, may prove effective. Simultaneously, the healthcare system, society, and caregivers must prepare for the increasing number of dementia patients. Improved diagnostics, new forms of therapy, and social innovations that support those who are affected and their relatives can help reduce the burden of dementia and its associated costs.

## 1. Introduction

Germany’s demographic development is characterized by declining birth rates and increasing life expectancy. Over the past 30 years, life expectancy at the age of 65 has increased by 3.1 years for women (from 18.0 to 21.1 years) and by 3.5 years for men (from 14.3 to 17.8 years) [1]. Consequently, more and more people – predominantly women – are reaching very high age; for example, the number of people aged 85 and older rose from 1.1 million in 1990 to 2.5 million in 2020, and is expected to at least double by 2070 [2]. The rising number and proportion of elderly people in the population is associated not only with an imbalance between the working and care-dependent population, but also with health-related challenges. Advanced age is one of the most important health risks [3], and dementia is one of the most common age-associated diseases. In 2019, about 1.7 million people with dementia lived in Germany [4] and forecasts indicate a future increase in their number up to 3.0 million by 2070. Against this background, the article presents a clinical overview of dementia, as well as its epidemiology, risk factors, preventive measures, and care requirements.

**Infobox 1 Dementia**

Dementia is not a part of normal aging, nor is it a disease in and of itself. Instead, it is a collection of symptoms with diverse causes, signs, severity levels, and courses of disease. It is essentially characterised by changes in the brain and is associated with memory loss and changes in one’s personality [5, 6].

**Figure 1:**
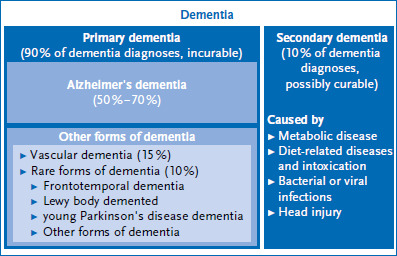
Forms of dementia Source: Figure based on [7–11]

Based on the underlying disease, a distinction can be made between primary and secondary dementia (Figure 1).

A total of 90 % of the dementia cases are primary dementia, which originate directly in the brain and are incurable. These include neurodegenerative forms, such as Alzheimer’s dementia, Parkinson’s disease dementia, Lewy body dementia or frontotemporal dementia. Non-neurodegenerative forms include vascular dementia, infectious dementia, such as sporadic Creutzfeldt-Jakob disease, or hereditary forms of dementia.

A total of 10 % of dementia cases are secondary dementia (secondary to other underlying diseases), and can, in principle, be cured [7]. Metabolic, nutritional or poisoning-related diseases (e.g. vitamin E deficiency, alcohol and drug consumption), bacterial or viral infections (e.g. HIV) or head injuries can cause them [7–9].

### 1.1 Forms of dementia

Alzheimer’s dementia (50 % – 70 %) and vascular dementia (15 %) are the most common forms. Rare forms of dementia, such as frontotemporal dementia, Lewy body dementia or dementia caused by a previous disease (e.g. Parkinson’s disease), comprise approximately 10 % of the cases [10, 11]. It is difficult to exactly determine the proportions of the various forms of dementia due to their varied origins and symptoms as well as the inconsistency of their diagnosis. Autopsy studies suggest that at the end of their lives, most people suffering from dementia have a combination of Alzheimer’s dementia and vascular dementia [12].

Normal cognitive ageing processes and mild cognitive impairment are not discussed in this article, although they are often considered to be precursors of dementia [13]. The focus is on senile dementia, which has its onset approximately at the age of 65 [11].

### 1.2 Course and symptoms

Most forms of dementia have a gradual onset. As a result, distinguishing between general cognitive ageing and early mild dementia is challenging. Defining the transition from normal cognitive ageing to the early stages of cognitive disease is difficult [13]. While individuals with normal cognitive ageing can compensate for the cognitive losses and can act independently [14], dementia is characterised by a more rapid, progressive decline in cognitive performance. This is apparent in areas such as orientation, communication skills, attention, and concentration [29, 30]. Moreover, almost all forms of dementia are accompanied by varying psychological well-being and behavioural changes, including depressive symptoms, anxiety, sleep disorders, delusions or aggression [9]. Depending on the specific form of dementia, other symptoms, such as speech disorders, mood swings, motor impairments or hallucinations may occur as well. The broad range of symptoms makes it difficult to accurately diagnose dementia and determine its severity. In addition, distinguishing the core psychological symptoms of dementia from those of other underlying diseases is difficult [9].

## 2. Methods

### 2.1 Data and data collection

It is common to use either population-based epidemiological surveys or health claims data from statutory health insurance companies to analyse dementia. Standardized neuropsychological tests are commonly used in epidemiological surveys to measure cognitive impairment and investigate the causes of diseases [15, 16]. The scientific use of these surveys has limitations due to their low case numbers, particularly when considering subgroups (e.g. by age, gender). There are also representativeness issues due to sample recruitment or different diagnostic procedures by physicians. Additionally, people who are in poor health and residents of care institutions are often excluded [17].

Health claims data are produced during the standard delivery of healthcare and nursing care. Physicians generate these data while billing their medical services to the healthcare insurer. Although health claims data are collected without any scientific intent, they permit tracking the complete disease and healthcare utilisation of a large number of individuals over time. This includes individuals residing in private households and nursing homes [18]. Diseases are usually recorded using the ICD-10 classification system. However, health claims often do not comprise clinical information and external validation of diagnoses is not possible. Furthermore, they are subject to legal changes.

The epidemiological measures of dementia in this article are based on anonymised claims data from the Allgemeine Ortskrankenkassen (AOKs), the largest statutory health insurance provider in Germany. This data covers approximately one third of the total population, which is equivalent to 26.8 million insured individuals. The AOK data includes payments made to certified physicians for outpatient care (according to § 295 para. 2 SGB V) and inpatient care (according to § 301 para. 1 SGB V). The following ICD-10 codes were used to register cases of dementia: G30, G31.0, G31.82, G23.1, F00, F01, F02, F03 and F05.1. This article does not distinguish between different types of dementia, as a significant proportion of diagnoses were for non-specific dementia (F03). Previous studies have indicated that AOK data is appropriate for estimating the prevalence, incidence, and trends in dementia over time [19].

### 2.2 Estimation of incidence and prevalence

Prevalence and incidence are the most important epidemiological measures for describing the extent of a disease. Prevalence refers to the proportion of current cases of a disease in a total population during a specific period of time. Incidence is the rate of new cases of a disease among the healthy population who are at risk of acquiring the disease within a specific timeframe [20]. The current prevalence estimates are based on a 2.2 % random sample of individuals aged 50 years and over, insured by AOK in the year 2014. Incidence estimates were produced by following individuals from the base year until 2019.

### 2.3 Forecasting the number of dementia patients

Forecasting models for predicting the future number of dementia patients take into account changes in population structure, life expectancy and the prevalence of dementia. Based on age-specific dementia prevalence (see 3.1 Prevalence and incidence) and the 15th Coordinated Population Projection of the Federal Statistical Office (variants: G2L2W2 – moderate increase in life expectancy, G2L3W2 – large increase in life expectancy [21]), we projected the number of dementia patients aged 65 and older in Germany until 2070. To achieve this, we used the moderate migration scenario for the population projections (W2) and two different scenarios for the development of dementia prevalence: The status quo scenario (S1.1 and S1.2) assumes a constant disease prevalence with increasing life expectancy, while the prevention scenario (S2.1 and S2.2) assumes a reduction in age-specific disease prevalences by 1 % per year.

### 2.4 Disease burden of dementia

The impact of dementia on population health was calculated using data from the BURDEN 2020 project at the Robert Koch Institute (RKI). To represent the burden of disease, the disability-adjusted life years (DALY) indicator was used. This indicator combines the years of life lost due to death (YLL, years of life lost, known as the mortality component) and the years lost due to health impairment (YLD, years lost due to disability, morbidity component) within the population [22]. YLL represent the gap between the disease-related age at death and the age-specific remaining life expectancy. YLD is derived by combining the disease prevalence with the disability weight, which indicates the degree of impairment. Consequently, one DALY equals one year of healthy life ‘lost’.

The AOK data were also used to calculate the YLD related to dementia in this article [22]. The cause of death statistics were used to calculate the YLL [22, 23]. The cause of death statistics show the basic illness (underlying cause of death) for each deceased person. However, there are also direct (the last illness that was directly fatal) and contributing causes of death [24]. Diseases such as heart attack or lung cancer are more directly associated with death than dementia. Therefore, these diseases are recorded more accurately as the underlying cause of death. In contrast, dementia is often regarded as a contributing cause of death, which means that dementia is not fully recorded in the cause of death statistics [25]. This, in turn, may result in an underestimation of the overall burden of disease caused by dementia.

## 3. Epidemiology of dementia

### 3.1 Prevalence and incidence

In 2014, the prevalence of dementia at age 65 and older was 10.3 % in Germany, and was 1.7 % in the group of 65 to 69 years of age. It rises exponentially with increasing age, doubling roughly every five to six years until the age of 80 to 84. From age 95, the prevalence stabilises at a high level (Table 1).

From 2014 to 2019, the incidence of dementia at age 65 and older was 2.4 new cases per 100 person-years. The incidence is characterised by an exponential increase with age (Table 1). In the age group of 65 to 69 years of age, there were 0.4 new diagnoses per 100 person-years in men and women combined. This number increases with age, up to 11.3 (men) and 11.9 (women) in the group of 95 years and older. Gender differences in the prevalence can be explained by higher mortality rates among men, which leave only the healthiest individuals to survive into old age.

### 3.2 Forecasts

**Figure 2:**
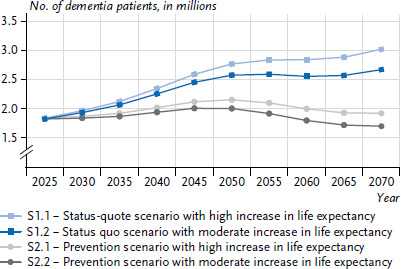
Forecast of the number of dementia patients aged 65 and older in Germany by 2070 (n = 83,504 women, n = 55,958 men) Source: Sample from health claims data of persons insured with AOK 2014 – 2019 and 15th Coordinated Population Projection of the Federal Statistical Office [21]

Previous forecasts up to 2060 indicate that there will be a decline in the number of dementia patients from around 2050, when the large baby boomer cohorts (born in the 1950s and early 1960s) will be 85 years of age or older [4]. Even when taking into account the increase in life expectancy, at this high age mortality is high and remaining life expectancy low [26], so that the contribution of this large cohort to the total prevalence at age 65 and older will get progressively smaller over time [26]. In the present status quo scenario, the number of dementia patients aged 65 and older will increase from an estimated 1.8 million in 2025 to 2.8 million (scenario S1.1) or 2.6 million (scenario S1.2) in 2055 (Figure 2). Thereafter, there will be a further sharp increase when, from 2055 onwards, the children of the baby boomers (born between 1980 and 1995) enter the age groups with high prevalence of dementia. By 2070, the number of dementia patients will increase to 3.0 million (S1.1) or 2.7 million (S1.2). If the prevalence of dementia can be reduced by an average of 1 % per year, the number of dementia patients would increase to a maximum of 2.2 million (S2.1) or 2.0 million (S2.2) in 2050. Subsequently, the number would drop again to 1.9 million (S2.1) or 1.7 million (S2.2) persons in 2070. Despite inherent uncertainty and various influencing factors, such as migration, pandemics, and changes in mortality patterns, projections for 2070 suggest that the number of dementia patients will still increase after the baby-boom generation has passed unless preventive measures are implemented or new therapies are developed.

### 3.3 Mortality

Dementia patients have higher death rates and a shorter life expectancy than those who are not afflicted by dementia. In a Swedish study, the average survival time of dementia patients is 4.8 years in the age group of 75 to 84 years of age and 3.8 years in the age group of 85 years and older [27].

The progressive health decline during the course of disease increases the risk of falling, multimorbidity and infections. Pneumonia, urinary tract infections, bone fractures or organ failure, for example, occur more frequently in persons with dementia, which increases their mortality risk. As a result, in the period from 2014 to 2017, dementia was the most common disease at the time of death among women aged 70 years or older and the fifth most common disease among men. In 2060, dementia will maintain this position among women and become the second most common disease at the time of death among men, as illustrated by a study for Germany based on AOK claims data for the years 2014 to 2017 [28].

### 3.4 Burden of disease

**Figure 3:**
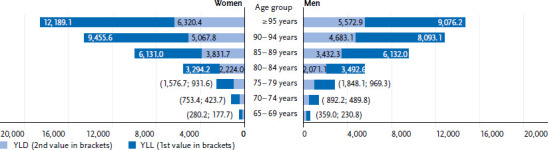
Burden of disease (DALY per 100,000 persons by YLL and YLD) of dementia with increasing age and by gender in 2017 Source: Robert Koch Institute, special analysis of the BURDEN 2020 study

The burden of disease increases sharply with age for both women and men. On average, 100,000 men 65 to 69 years of age lost 590 years of life due to dementia (health impairment and death, DALY), compared to 458 years in women of the same age. At age 95, the number of years lost to dementia (DALY) increases to 18,509 years per 100,000 women and 14,649 years per 100,000 men (Figure 3). It is also evident – despite the potential under-reporting of dementia as a cause of death, as only the underlying cause of death is taken into account for the calculation (see Chapter 2.4) – that a larger share of the burden of disease is attributable to deaths due to dementia (YLL). The proportion due to dementia-related disability (YLD) is lower for all age groups. Deaths account for almost two-thirds of the disease burden in individuals aged 65 and older (women: 62.3 %, men: 63.8 %), while health impairment measures approximately one-third (women: 37.7 %, men: 36.2 %).

**Figure 4:**
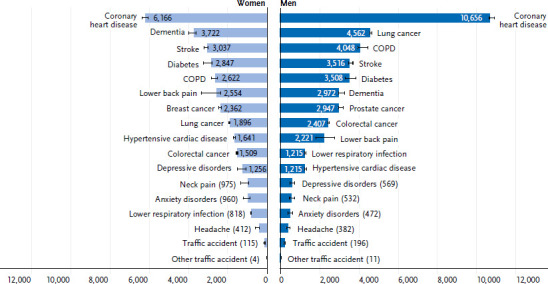
Total burden of disease (DALY per 100,000 persons) for the most common causes of burden of disease by gender at age 65+ Source: Robert Koch Institute, special analysis of the BURDEN 2020 study (ranking based on selected important causes of burden of disease, see [22])

In 2017, the dementia-related burden of disease in the group of 65 years of age and older was 3,722 DALY per 100,000 persons for women and 2,972 DALY per 100,000 persons for men (Figure 4). When comparing the burden of disease for different illnesses, coronary heart disease resulted in the highest number of years of life lost for both women (6,166 DALY) and men (10,656 DALY). Dementia ranked second for women (3,722 DALYs) and sixth for men (2,972 DALYs) among the major causes of burden of disease selected herein (Figure 4).

### 3.5 Costs

Due to the high need for care and the high multi-morbidity, dementia is one of the most expensive group of diseases from the age of 65. The direct medical and non-medical costs of illness borne by the health and care insurance companies make up only a small fraction of the total costs. Of greater importance are the indirect costs for nursing and care: A total of 75 % to 80 % of the illness costs of dementia are accounted for by lost income and tax losses due to informal care [17, 29]. Informal care is usually provided without payment by family care givers, friends, neighbours or other non-trained care givers in the domestic environment of the person in need of care [29]. It is an important cost factor especially when care givers reduce or terminate their employment to be able to provide care [30].

Because the costs associated with dementia occur in a variety of settings and are not fully captured, such as lost income for caregivers, they must be estimated. These estimates are based on assumptions and therefore vary to some extent. According to a comprehensive meta-analysis, the direct annual cost of dementia for people aged 65 and older in 2016 was approximately € 34 billion for health and care insurance companies, including expenditures on, prevention, treatment, and rehabilitation, and an additional € 73 billion in costs to society as a whole, mainly due to informal care. The costs of informal care only consider services that result in a loss of productivity, for example when employed persons reduce their working hours to provide care. It is expected that these costs will rise to € 90 billion (direct costs) and € 195 billion (indirect costs) by 2060. The average annual direct cost per dementia patient is about € 20,658 for the health insurance and € 44,659 for society as a whole. Compared to individuals without dementia, the yearly excess costs per person are € 11,205 for the health insurance and € 33,188 for the society as a whole [31].

## 4. Risk factors and prevention

### 4.1 Risk factors

**Figure 5:**
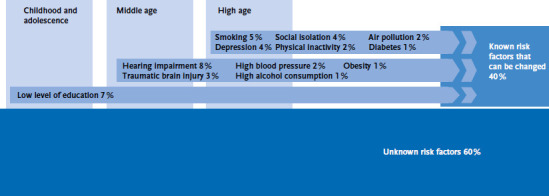
Risk factors of dementia; percentages illustrate the possible decrease in dementia prevalence if the corresponding risk factor were eliminated Source: own presentation according to [32]

Dementia develops over the course of decades and sometimes manifests long before symptoms become apparent. The diverse forms of dementia also contribute to the fact that around 60 % of dementia cases are still unexplained in terms of their causes, as indicated by the *Lancet Commission on dementia prevention, intervention, and care*. This implies that a substantial portion of the factors that increase the risk of dementia is still unknown [32]. Around 40 % of the dementia cases are therefore attributable to risk factors over the entire life course and could thus be prevented (Figure 5). An international meta-analysis identified 26 risk factors for dementia [33]. Notably, the risk factors identified for Europe largely overlap with those highlighted by the Lancet Commission. Apart from age and gender, social, lifestyle and health-related factors, e.g. other diseases, are important dementia risk factors [32]. They collectively contribute to the risk of dementia and provide crucial foundations for preventive measures.

Educational level is an important predictor of overall health and dementia, with individuals with high education having a lower risk of dementia. This association has been attributed to two indirect pathways: better educated individuals often have healthier lifestyles and education increases cognitive reserve capacity, which allows for compensation of the cognitive decline [34, 35].

In middle age (45 – 65 years), hearing loss has the greatest impact of all identified risk factors: Approximately 8 % of dementia cases could be prevented [32]. Presumably, secondary effects, such as depression or social isolation, may explain this association, at least in part [36]. The *Lancet Commission on dementia prevention, intervention, and care* also identified traumatic brain injury, hypertension, heavy alcohol consumption and obesity as relevant factors in midlife [32]. Studies in Germany corroborate all these risk factors [37–39].

At advanced age, about 5 % of the dementia cases are due to tobacco use [32], while another 4 % each are due to depression and social isolation. Physical inactivity, which is closely linked to risk factors mentioned above, explains another 2 % of all dementia cases. Even some limitations in mobility, for example disturbances of the gait pattern [40] or injuries to the lower extremities [41], might be associated with an increased risk of dementia. Diabetes accounts for 1 % of all dementia cases, with numerous studies showing the significance of this metabolic disease in the onset of dementia [39, 42, 43]. Environmental factors, measured as air pollution, have also been demonstrated to affect cognitive performance [44]. Structural characteristics such as regional differences in wealth can also contribute to differences in the risk of dementia [35]. One study for Germany shows that about 30 % of Alzheimer’s dementia cases were attributed to seven risk factors, namely diabetes, high blood pressure and obesity in middle age, depression, physical inactivity, smoking and low educational level [45].

Other studies showed that cardiovascular diseases such as strokes and coronary heart disease, as well as elevated blood cholesterol levels (hypercholesterolaemia) [39, 46, 47] may increase the risk of dementia. Also drug therapies used for other diseases, for instance, antipsychotics, urological drugs, or psychostimulants [37] are linked to a higher risk of dementia. On the other hand, some drugs may even have a protective effect and might be associated with a lower risk of dementia [48]. According to recent research, another factor that could influence cognitive health might be the interaction between the central nervous system and the gut microbiome, although the underlying mechanisms are not yet fully understood [49].

It should be borne in mind that many of the effects may be indirect as well as direct, and that risk factors may interact. In particular, the presence of multiple risk factors adversely affects the risk of dementia [50].

### 4.2 Prevention

Effective dementia prevention thus requires above all the reduction of lifestyle-related impairments and pre-existing conditions [51]. A healthy lifestyle, including a balanced diet, a healthy body weight, avoiding alcohol and smoking, can achieve this. Promoting physical activity is especially important in this context because it accounts for about two out of ten Alzheimer’s dementia cases in Germany, making it one of the most influential factors in reducing the risk of dementia [45]. Furthermore, reducing and diagnosing dementia-related illnesses, such as hearing loss, depression, high blood pressure, and diabetes, early and treating them appropriately can help prevent dementia. It should be noted that some studies presume complete elimination of risk factors [32] or a strong decrease in risk factor prevalence [45]. Thus the actual prevention potential might be overestimated. Using the example of type 2 diabetes in the age group of 75 and older, a study based on AOK data from 2014 shows that a 1 % reduction in this disease alone could reduce the number of dementia cases by about 30,000 in 2040. If the incidence of dementia among diabetics was successfully reduced by just 1 %, 220,000 dementia diagnoses could be prevented [43]. International studies highlight that promoting stimulating cognitive, physical, and social activities could potentially have a strong prevention impact [52, 53].

At the societal level, social inclusion, reducing environmental stress and regional economic inequality, as well as increasing educational attainment have shown promise in the prevention of dementia. The latter also includes formal education as well as maintaining cognitive performance in old age, e.g. through lifelong learning or memory training. Combining multiple protective factors and reducing risk factors over the course of life simultaneously has a particularly beneficial effect [54]. Almost all protective factors have in common that they can reduce both the risk of dementia and the risk of severe disease progression [47]. Since even mild cognitive impairment can be associated with an increased risk of dementia [55], early diagnosis and treatment of dementia is also proving to be an important element in the delay of the severity progression of the disease [56, 57].

## 5. Care

### 5.1 Care options

While the development of new antibody therapies is raising cautious hope for a therapy for dementia [58, 59], at this time there are no drugs that can effectively prevent or slow down the disease. Therefore, the focus is on measures for long-term care as well as for improving the quality of life, for maintaining social, cognitive and everyday abilities and for delaying the progression of the disease. In the mild stage of the disease, non-drug therapies such as cognitive training, occupational and physiotherapy and psychosocial interventions can promote well-being and maintenance of function. This is one of the reasons why a number of innovative life management services for people with dementia have been developed in recent years.

### 5.2 Provision of care

A total of 90 % of dementia patients require end-of-life care due to the loss of function caused by the disease. Therefore, dementia is one of the main reasons for requiring care [29]. Coping with the need for care is predominantly a private and familial matter: the majority of the dementia patients live at home, and about two thirds of them receive informal care from close relatives [31, 60].

This form of care applies mainly to younger dementia patients or those in an early, mild stage of the disease. Most dementia patients prefer family care [61] and it is an essential pillar of the provision of care in Germany. However, informal care is also associated with high societal costs and health, mental and financial burdens for the care givers, typically spouses, children or other family members. Women bear a significantly higher burden of care [29, 31, 62, 63].

People with dementia require care for longer periods and have higher demands and burdens than those without dementia due to core and psychosocial symptoms [64]. Professional outpatient or inpatient care is usually only sought when the disease becomes more severe [65]. There is already an imbalance between the demand for and supply of care, which is expected to increase in the future. According to forecasts, the number of people with statutory health insurance who are in need of long-term care will increase from about 3.3 million in 2017 to about 5.1 million in 2060 [66], and the number of the recipients of informal care increasing from 3.1 million to 4.1 million [67]. Professional outpatient care is expected to increase from 0.9 to up to 1.4 million persons in need of care, and institutional care is expected to increase from 0.8 to up to 1.3 million persons [67]. These figures will peak in 2050, but could be significantly reduced by improvements in morbidity, mortality and risk factor development. To meet the growing demand for care, the number of professional care workers in the outpatient and inpatient sectors would need to increase by about 394,000 persons (from 586,000 in 2017 to 980,000 in 2060) [66]. However, the increasing demand for care is offset by a decline in the available care workers of about 20 %, and the informal care potential in the population, i.e. the number of potential informal care givers, is also expected to increase less strongly than the demand for care [67].

**Infobox 2 National dementia strategy**

The recommendations for action for the global handling of dementia contained in the *Global action plan on the public health response to dementia* 2017 – 2025 of the WHO have been taken into account for Germany in the National Dementia Strategy (www.nationale-demenzstrategie.de). The strategy comprises a total of 162 measures and 27 sub-goals in four fields of action:

► Developing and establishing structures for social participation of people with dementia at their place of residence
► Providing support to people with dementia and their relatives
► Advancing medical and nursing care for people with dementia
► Promoting excellent research on dementia

The 162 measures of the National Dementia Strategy are designed to contribute to improving the living situation, everyday life, social participation and healthcare and nursing care given to dementia patients and their relatives. This includes, for example, new social spaces and mobility concepts, raising public awareness, new housing concepts, (expanded) counselling services for dementia patients and their relatives, strengthening and improving care, and in-depth dementia research [68].

### 5.3 Recent programmes and offers for care and support

Various services that are sensitive and responsive to the needs of individuals with dementia and that complement therapy and nursing care have been developed recently. These services can enhance the well-being of dementia patients by preserving their participation and autonomy. They are often regional or temporary social structures and have a significant impact on the everyday life, e.g. dementia gardens, animal-assisted interventions, musical and artistic activities or sports groups [69]. Digital or technical aids, on the one hand in the form of online self-help groups or dementia podcasts, on the other hand as support in home care, e.g. in the form of tracking devices or security measures such as cooker detectors or key finders, can promote the exchange between those afflicted as well as their integration into the non-afflicted society. Such measures can also delay the need for institutional care [69, 70].

Residential projects also enable people with severe illness to live together independently, within a secure environment outside of inpatient or nursing homes. Residential groups, including those tailored to specific groups such as dementia patients with a migration history or homosexual dementia patients, promote social interaction and have been linked with enhanced quality of life and cost efficiency [71].

Local dementia networks are a cooperation of interdisciplinary medical, nursing and social services that provide a networked spectrum ranging from counselling and diagnostics to therapeutic support. As such, they partly enable holistic care and create an interface for dementia patients receiving outpatient care, their relatives and other actors [72]. Studies show the effectiveness of these networks, e.g. with regard to better pharmacological and medical care [73].

## 6. Conclusion and outlook

Dementia is a significant health issue in Germany and poses a major challenge not only for individuals, but also for society, nursing care and medical care. This challenge is expected to continue to grow in the upcoming decades due to demographic ageing, also depending on medical and health developments. Short-term migratory movements may affect the number of future dementia patients, even though large migratory flows are less frequent in older age groups. Since there is no cure for dementia at this time, even good prevention could at best compensate for the effects of the progressive ageing of society on the occurrence of dementia. Consequently, the health and care system, as well as society, should develop solutions that meet the needs of the increasing number of dementia patients.

Efficient and comprehensive dementia diagnosis, treatment, care and support are crucial for mitigating the impact of dementia on individuals and the society as a whole [51]. Medical care for dementia patients is based on four pillars affecting the disease’s progression and impact: early diagnosis, thorough symptom and impairment assessment, precise staging and monitoring, and tailoring of therapies [47]. Diagnosis, also with a view to the severity of the disease, is often inaccurate due to the broad and unspecific clinical symptoms [47] – which means that treatment options cannot be fully exploited. Dementia patients usually receive comprehensive medical care from neurologists and general practitioners, but diagnostic work-ups in the early stages of the disease and interdisciplinary treatment have so far been subject to limitations [30, 74]. Approaches for further development are therefore evident in integrated care and in improving basic knowledge about dementia that is conveyed in medical and nursing training [75]. However, this requires a higher utilisation by those afflicted at an early stage, when early and unspecific symptoms become manifest [57].

Due to the reduced informal and formal care potential and the increasing demand for care, changes in the care sector are still necessary. Informal care givers need support [51], as legal, social, emotional and financial services can alleviate the burdens of care giving. Telemonitoring and telemedicine are appropriate for improving the medical monitoring and effectively assessing the progress if disease, even in a home environment. With regard to institutional care, alternative care settings, such as innovative forms of housing or technical aids, have been shown to improve the conditions for both care givers and dementia patients [69, 70].

However, dementia is not solely a medical problem, but also a societal concern. This relates to the growing demand for informal care as well as the public visibility of dementia patients and their social integration. The stigmata and fears associated with the disease among the population and those afflicted contribute to social exclusion [76]. What seems desirable in this regard is greater acceptance of and sensitivity for the disease and a broader general understanding of dementia in society [51], as well as early education about the course of the disease and possible encountering strategies between those who are afflicted and those who are not. This could reduce uncertainties and ambiguities experienced by patients and their relatives, especially at the onset of the disease [68]. In the further course of disease, a dementia-sensitive environment is needed, that not only recognizes the behavioural changes and communicative, motoric-cognitive impairments associated with the disease, but also provides a supportive and safe environment [68, 69]. Both factors could help maintain the autonomy and quality of life, and thus also reduce the progression of disease and dependency on care. Civil society initiatives can help address these needs and may serve as an essential supplementary approach [69]. Studies have shown the efficacy of such innovations for dementia patients [70, 71]. However, certain groups, such as dementia patients in structurally weak areas, those with a migration history, or in an advanced stage of the disease, may not be adequately reached by these services [69]. Therefore, it is important to establish existing services throughout Germany and in a structured manner, and to promote new services for specific target groups [68, 69].

At the societal and individual level, there is still a need to raise awareness of the risk factors of dementia. Living a healthy lifestyle has potential to decrease not only the risk of dementia but also the likelihood of other illnesses [77]. It is essential to not only consciously evaluate one’s lifestyle but also minimize health disparities [77]. By adopting preventive measures, the healthcare system could reduce the cost of treatment and care relating to dementia while providing benefits.

Although dementia and the afflicted individuals have become more present in society in recent years, the public remains unaware of the available services and strategies. The National Dementia Strategy, which considers the various actors and provides a framework for action, is a comprehensive set of measures that was introduced for the first time in Germany. The promotion of in-depth dementia research within the National Dementia Strategy, for example through measures for basic and epidemiological research, can provide important new insights into the various forms of dementia, their risk factors and their impact [68]. However, representative data are currently lacking to adequately represent the diversity of the populations. Minorities, such as ethnic or gendered minorities are only rarely taken into consideration [78]. The health claims data used herein are also subject to bias. For example, those insured by the AOK have a lower socioeconomic status and a higher morbidity rate on average compared to others with statutory or private insurance [79]. Furthermore, the health claims data are primarily compiled for billing purposes, rather than for epidemiological analyses. The documented diagnoses thus only partially reflect the epidemiological disease development at the population level, because they only record persons who have consulted a doctor and had a diagnosis made. On the one hand, coding errors and false-positive diagnoses can occur, on the other hand, cases of dementia in their early stages are sometimes not diagnosed at all [28, 43]. To plan effectively, gain better insights into dementia’s development, identify options for action, and provide efficient care to dementia patients collecting more clinical, epidemiological, and population-based routine and survey data is necessary for the future. Considering recent research directions, additional information, such as biomarkers, should be included [78]. From the point of view of research and in the interest of patients, greater transparency and availability of already existing data sources as well as the integration of new data sources, for example information from electronic patient records, are desirable and urgently needed.

## Key statement

- Population ageing contributes to an ongoing increase in the number of dementia patients.
- Cognitive, motor, and psychosocial symptoms associated with dementia reduce the quality of life and impair the ability to live autonomously.
- Dementia is linked to substantial costs to society because of the considerable requirement for care.
- Lifestyle factors play a critical role in determining the risk of dementia and may offer strategies for prevention.
- Society, healthcare, medicine, and politics must develop viable solutions to address the increase in the number of dementia patients, including finding suitable care options.

## Figures and Tables

**Table 1:** Age-specific prevalence of dementia per 100 persons in 2014 and age-specific incidence of dementia per 100 person-years in 2014 – 2019 (from age 65), Germany (n = 83,504 women, n = 55,958 men) Source: Sample from health claims data of all persons insured with AOK 2014 – 2019

Age group	Prevalence			Incidence		
	Women	Men	Total	Women	Men	Total
65 – 69 years	1.6	1.9	1.7	0.4	0.4	0.4
70 – 74 years	3.7	4.1	3.9	1.0	1.1	1.1
75 – 79 years	7.4	7.9	7.6	2.0	2.3	2.2
80 – 84 years	15.6	14.5	15.2	3.9	3.6	3.8
85 – 89 years	25.0	21.2	23.9	6.2	6.2	6.2
90 – 94 years	36.5	28.9	35.0	9.5	9.0	9.4
≥ 95 years	43.5	33.7	42.1	11.9	11.3	11.8
**Total**	**11.8**	**8.2**	**10.3**	**2.7**	**2.1**	**2.4**

## References

[1] Statistisches Bundesamt (2022) Sterbetafeln 2019/2021. Statistischer Bericht, Wiesbaden. https://www.destatis.de/DE/Themen/Gesellschaft-Umwelt/Bevoelkerung/Sterbefaelle-Lebenserwartung/Publikationen/Downloads-Sterbefaelle/statistischer-bericht-sterbetafeln-5126207217005.html (As at 24.08.2023)

[2] Statistisches Bundesamt (2022) Bevölkerungsentwicklung bis 2070 in Deutschland. Vorausberechneter Bevölkerungsbestand, Wiesbaden. https://www.destatis.de/DE/Themen/Gesellschaft-Umwelt/Bevoelkerung/Bevoelkerungsvorausberechnung/_inhalt.html (As at 24.08.2023)

[3] Böhm K Tesch-Römer C Ziese T (Hrsg) (2009) Gesundheit und Krankheit im Alter. Beiträge zur Gesundheitsberichterstattung des Bundes. Robert Koch-Institut, Berlin. https://edoc.rki.de/handle/176904/3220 (As at 24.08.2023)

[4] GBD 2019 Dementia Forecasting Collaborators (2022) Estimation of the global prevalence of dementia in 2019 and forecasted prevalence in 2050: an analysis for the Global Burden of Disease Study 2019. Lancet Public Health 7:e105–e125. https://doi.org/10.1016/S2468-2667(21)00249-8 (As at 08.08.2023) 34998485 10.1016/S2468-2667(21)00249-8 PMC8810394

[5] Europäische Union (2005) Rare forms of dementia. Final report of a project supported by the Community Rare Diseases Programme 2000 – 2002. https://ec.europa.eu/health/archive/ph_threats/non_com/docs/raredementias_en.pdf (As at 08.08.2023)

[6] Eurostat (2022) Mental health and related issues statistics. https://ec.europa.eu/eurostat/statistics-explained/index.php?title=Mental_health_and_related_issues_statistics (As at 08.08.2023)

[7] Stoppe G Staedt J (2002) Potenziell behebbare Demenzen. In: Beyreuther K Einhäupl KM Förstl H. (Hrsg) Demenzen – Grundlagen und Klinik. Georg Thieme Verlag, Stuttgart, P. 414–431

[8] Gupta S Fiertag O Warner J (2009) Rare and unusual dementias. Adv. psychiatr. treat 15:364–371. https://doi.org/10.1192/apt.bp.107.003558 (As at 08.08.2023)

[9] Kastner U Schraut V Löbach R (2022) Handbuch Demenz. Fachwissen für Pflege und Betreuung, 5. Auflage. Urban & Fischer in Elsevier, München

[10] Kilimann I Teipel S (2013) Alzheimer-Krankheit. In: Bartsch T Falkai P (Hrsg) Gedächtnisstörungen. Springer, Berlin, Heidelberg, P. 239–263

[11] Winblad B Amouyel P Andrieu S. (2016) Defeating Alzheimer‘s disease and other dementias: a priority for European science and society. Lancet Neurol 15:455–532. https://doi.org/10.1016/S1474-4422(16)00062-4 (As at 08.08.2023) 26987701 10.1016/S1474-4422(16)00062-4

[12] Rabinovici GD Carrillo MC Forman M. (2017) Multiple comorbid neuropathologies in the setting of Alzheimer’s disease neuropathology and implications for drug development. Alzheimers Dement (NY) 3:83–91. https://doi.org/10.1016/j.trci.2016.09.002 (As at 08.08.2023) 10.1016/j.trci.2016.09.002 PMC5651346 29067320

[13] Reupke I (2010) Psychiatrische Erkrankungen im Alter – Vaskuläre Demenzen. Brennpunkt Demenz. https://docplayer.org/14103583-Psychiatrische-erkrankungen-im-alter-vaskulaere-demenzen-brennpunkt-demenz-06-november-2010.html (As at 08.08.2023)

[14] Larson EB Kukull WA Katzman RL (1992) Cognitive impairment: dementia and Alzheimer‘s disease. Annu Rev Public Health 13(1):431–449 1599598 10.1146/annurev.pu.13.050192.002243

[15] Launer LJ (2011) Counting dementia: There is no one ‘best’ way. Alzheimers Dement 7:10–14. https://doi.org/10.1016/j.jalz.2010.11.003 (As at 08.08.2023) 21255740 10.1016/j.jalz.2010.11.003 PMC3133460

[16] Mitchell AJ Malladi S (2010) Screening and case finding tools for the detection of dementia. Part I: evidence-based meta-analysis of multidomain tests. Am J Geriatr Psychiatry 18:759–782. https://doi.org/10.1097/JGP.0b013e3181cdecb8 (As at 08.08.2023) 20808118 10.1097/JGP.0b013e3181cdecb8

[17] Doblhammer G Schulz A Steinberg J. (2012) Demografie der Demenz. 1. Aufl. Verlag Hans Huber: Programmbereich Gesundheit. Huber, Bern

[18] Swart E Ihle P (2014) Sekundärdatenanalyse: Aufgaben und Ziele. In: Swart E Ihle P Gothe H. (Hrsg) Routinedaten im Gesundheitswesen. Handbuch Sekundärdatenanalyse: Grundlagen, Methoden und Perspektiven. 2., vollst. überarb. und erw. Aufl. Huber, Bern, P. 16–18

[19] Doblhammer G Fink A Zylla S. (2015) Compression or expansion of dementia in Germany? An observational study of short-term trends in incidence and death rates of dementia between 2006/07 and 2009/10 based on German health insurance data. Alzheimers Res Ther 7:66. https://doi.org/10.1186/s13195-015-0146-x (As at 08.08.2023) 26537590 10.1186/s13195-015-0146-x PMC4634148

[20] Jessen F (Hrsg) (2018) Handbuch Alzheimer-Krankheit: Grundlagen – Diagnostik – Therapie – Versorgung – Prävention. De Gruyter, Berlin, Boston. https://doi.org/10.1515/9783110411003 (As at 23.08.2023)

[21] Statistisches Bundesamt (2022) 15. koordinierte Bevölkerungs-vorausberechnung – Deutschland. Berichtszeitraum 2021–2070, Wiesbaden. https://www.destatis.de/DE/Themen/Gesellschaft-Umwelt/Bevoelkerung/Bevoelkerungsvorausberechnung/begleitheft.html (As at 24.08.2023)

[22] Porst M von der Lippe E Leddin J. (2022) The burden of disease in Germany at the national and regional level. Results in terms of disability-adjusted life years (DALY) from the BURDEN 2020 study. Dtsch Arztebl Int 119:785–792. https://doi.org/10.3238/arztebl.m2022.0314 (As at 08.08.2023) 36350160 10.3238/arztebl.m2022.0314 PMC9902892

[23] Wengler A Porst M Anton A. Ergebnisbericht BURDEN 2020. Die Krankheitslast in Deutschland und seinen Regionen. Grundlagen einer umfassenden Planung im Gesundheitswesen. https://innovationsfonds.g-ba.de/downloads/beschluss-dokumente/395/2023-03-01_BURDEN-2020_Ergebnisbericht.pdf (As at 17.08.2023)

[24] Bundesinstitut für Arzneimittel und Medizinprodukte (2014) Todesursachen in der Todesbescheinigung. Eine kurze Anleitung. https://www.bfarm.de/SharedDocs/Downloads/DE/Kodiersysteme/TU/totenscheinanleitung.html (As at 22.08.2023)

[25] Küppers L Ritz-Timme S Hartung B (2021) Died from or with dementia? The relationship between dementia and cause of death. Dtsch Med Wochenschr 146:677–682. https://doi.org/10.1055/a-1380-1436 (As at 08.08.2023) 33957690 10.1055/a-1380-1436

[26] Statistisches Bundesamt (2019) Bevölkerung im Wandel. Annahmen und Ergebnisse der 14. koordinierten Bevölkerungsvorausberechnung, Wiesbaden. https://www.destatis.de/DE/Presse/Pressekonferenzen/2019/Bevoelkerung/bevoelkerung-uebersicht.html (As at 24.08.2023)

[27] Rizzuto D Bellocco R Kivipelto M. (2012) Dementia after age 75: survival in different severity stages and years of life lost. Curr Alzheimer Res 9:795–800. https://doi.org/10.2174/156720512802455421 (As at 08.08.2023) 22299618 10.2174/156720512802455421

[28] Doblhammer G Fritze T Reinke C. (2022) Can dementia become the most prevalent disease at the time of death in Germany? Projections up to the year 2060 for the five most important diseases at the time of death. Population Ageing 15:523–540. https://doi.org/10.1007/s12062-022-09365-7 (As at 08.08.2023)

[29] Laporte Uribe F Heinrich S Wolf-Ostermann K. (2017) Caregiver burden assessed in dementia care networks in Germany: findings from the DemNet-D study baseline. Aging Ment Health 21:926–937. https://doi.org/10.1080/13607863.2016.1181713 (As at 08.08.2023) 27171484 10.1080/13607863.2016.1181713

[30] Michalowsky B Thyrian JR Eichler T. (2016) Economic analysis of formal care, informal care, and productivity losses in primary care patients who screened positive for dementia in Germany. J Alzheimers Dis 50:47–59. https://doi.org/10.3233/JAD-150600 (As at 08.08.2023) 26639964 10.3233/JAD-150600

[31] Michalowsky B Kaczynski A Hoffmann W (2019) The economic and social burden of dementia diseases in Germany – A metaanalysis. Bundesgesundheitsbl 62:981–992. https://doi.org/10.1007/s00103-019-02985-z (As at 08.08.2023) 10.1007/s00103-019-02985-z 31297549

[32] Livingston G Huntley J Sommerlad A. (2020) Dementia prevention, intervention, and care: 2020 report of the Lancet Commission. Lancet 396:413–446. https://doi.org/10.1016/S0140-6736(20)30367-6 (As at 08.08.2023) 32738937 10.1016/S0140-6736(20)30367-6 PMC7392084

[33] Anstey KJ Ee N Eramudugolla R. (2019) A Systematic review of meta-analyses that evaluate risk factors for dementia to evaluate the quantity, quality, and global representativeness of evidence. J Alzheimers Dis 70:S165–S186. https://doi.org/10.3233/JAD-190181 (As at 08.08.2023) 31306123 10.3233/JAD-190181 PMC6700718

[34] Meng X D‘Arcy C (2012) Education and dementia in the context of the cognitive reserve hypothesis: a systematic review with meta-analyses and qualitative analyses. PLoS One 7:e38268. https://doi.org/10.1371/journal.pone.0038268 (As at 08.08.2023) 22675535 10.1371/journal.pone.0038268 PMC3366926

[35] Kreft D Doblhammer G (2022) Sex and Gender Differences in Environmental Influences on Dementia Incidence in Germany, 2014 – 2019: An Observational Cohort Study Based on Health Claims Data. J Alzheimers Dis 87:223–237. https://doi.org/10.3233/JAD-215030 (As at 08.08.2023) 35275528 10.3233/JAD-215030 PMC9198799

[36] Fritze T Teipel S Óvári A. (2016) Hearing impairment affects dementia incidence. An analysis based on longitudinal health nlaims data in Germany. PLoS One 11:e0156876. https://doi.org/10.1371/journal.pone.0156876 (As at 08.08.2023) 27391486 10.1371/journal.pone.0156876 PMC4938406

[37] Reinke C Doblhammer G Schmid M. (2023) Dementia risk predictions from German claims data using methods of machine learning. Alzheimers Dement 19:477–486. https://doi.org/10.1002/alz.12663 (As at 08.08.2023) 35451562 10.1002/alz.12663

[38] Schrader J Lüders S (2016) Hypertonie und die Folgen für kognitive Funktionsstörungen und Demenz. CardioVasc 16:49–56. https://doi.org/10.1007/s15027-016-0744-y (As at 08.08.2023)

[39] Booker A Jacob LE Rapp M. (2016) Risk factors for dementia diagnosis in German primary care practices. Int Psychogeriatr 28:1059–1065. https://doi.org/10.1017/S1041610215002082 (As at 08.08.2023) 26744954 10.1017/S1041610215002082

[40] Verghese J Lipton RB Hall CB. (2002) Abnormality of gait as a predictor of non-Alzheimer‘s dementia. N Engl J Med 347:1761–1768. https://doi.org/10.1056/NEJMoa020441 (As at 08.08.2023) 12456852 10.1056/NEJMoa020441

[41] Zhou Y Putter H Doblhammer G (2016) Years of life lost due to lower extremity injury in association with dementia, and care need: a 6-year follow-up population-based study using a multi-state approach among German elderly. BMC Geriatr 16:9. https://doi.org/10.1186/s12877-016-0184-7 (As at 08.08.2023) 26758623 10.1186/s12877-016-0184-7 PMC4710990

[42] Reinke C Buchmann N Fink A. (2022) Diabetes duration and the risk of dementia: a cohort study based on German health claims data. Age Ageing 51. https://doi.org/10.1093/ageing/afab231 (As at 08.08.2023) 10.1093/ageing/afab231 PMC8753043 34923587

[43] Fink A Doerre A Demuth I. (2022) Potential of prevention strategies for the modifiable risk factor type 2 diabetes with relation to the future number of dementia patients in Germany – a multi-state projection through 2040. BMC Neurol 22:157. https://doi.org/10.1186/s12883-022-02682-6 (As at 08.08.2023) 35468764 10.1186/s12883-022-02682-6 PMC9040288

[44] Aretz B Janssen F Vonk JM. (2021) Long-term exposure to fine particulate matter, lung function and cognitive performance: A prospective Dutch cohort study on the underlying routes. Environ Res 201:111533. https://doi.org/10.1016/j.envres.2021.111533 (As at 08.08.2023) 34153335 10.1016/j.envres.2021.111533

[45] Luck T Riedel-Heller SG (2016) Prevention of Alzheimer‘s dementia in Germany: A projection of the possible potential of reducing selected risk factors. Nervenarzt 87:1194–1200. https://doi.org/10.1007/s00115-015-0045-1 (As at 08.08.2023) 26781245 10.1007/s00115-015-0045-1

[46] Doblhammer G Fritze T Teipel S (2014) Spatial patterns of dementia prevalence and its vascular risk factors in Germany. In: Doblhammer G (Hrsg) Health among the elderly in Germany. New evidence on disease, disability and care need. Barbara Budrich, Opladen, Berlin, Toronto, P. 51–68

[47] Maier W Jessen F Schneider F. (2010) S3-Leitlinie »Demenzen« Langversion (B). In: Deutsche Gesellschaft für Psychiatrie, Psychotherapie und Nervenheilkunde (DGPPN), Deutsche Gesellschaft für Neurologie (DGN) (Hrsg) Diagnose- und Behandlungsleitlinie Demenz. Springer Berlin Heidelberg, Berlin, Heidelberg, P. 9–72

[48] Nerius M Haenisch B Gomm W. (2020) Glucocorticoid Therapy is Associated with a Lower Risk of Dementia. J Alzheimers Dis 73:175–183. https://doi.org/10.3233/JAD-190444 (As at 08.08.2023) 31771051 10.3233/JAD-190444

[49] Connell E Le Gall G Pontifex MG. (2022) Microbial-derived metabolites as a risk factor of age-related cognitive decline and dementia. Mol Neurodegener 17:43. https://doi.org/10.1186/s13024-022-00548-6 (As at 08.08.2023) 35715821 10.1186/s13024-022-00548-6 PMC9204954

[50] Grande G Marengoni A Vetrano DL. (2021) Multimorbidity burden and dementia risk in older adults: The role of inflammation and genetics. Alzheimers Dement 17:768–776. https://doi.org/10.1002/alz.12237 (As at 08.08.2023) 33403740 10.1002/alz.12237 PMC8247430

[51] World Health Organization (2017) Global action plan on the public health response to dementia 2017 – 2025. https://www.who.int/publications/i/item/9789241513487 (As at 24.08.2023)

[52] Middleton LE Yaffe K (2010) Targets for the prevention of dementia. J Alzheimers Dis 20:915–924. https://doi.org/10.3233/JAD-2010-091657 (As at 08.08.2023) 20413867 10.3233/JAD-2010-091657

[53] Mangialasche F Kivipelto M Solomon A. (2012) Dementia prevention: current epidemiological evidence and future perspective. Alzheimers Res Ther 4:6. https://doi.org/10.1186/alzrt104 (As at 08.08.2023) 22339927 10.1186/alzrt104 PMC3471409

[54] Escher C Jessen F (2019) Prevention of cognitive decline and dementia by treatment of risk factors. Nervenarzt 90:921–925. https://doi.org/10.1007/s00115-019-0759-6 (As at 08.08.2023) 31440769 10.1007/s00115-019-0759-6

[55] Reischies FM Bürker BS (2005) Leichte Kognitive Störung und Mild Cognitive Impairment. Z Gerontol Geriatr 18:203–225. https://doi.org/10.1024/1011-6877.18.4.203 (As at 08.08.2023)

[56] Arevalo-Rodriguez I Smailagic N Roqué-Figuls M. (2021) Mini-Mental State Examination (MMSE) for the early detection of dementia in people with mild cognitive impairment (MCI). Cochrane Database Syst Rev 7:CD010783. https://doi.org/10.1002/14651858.CD010783.pub3 (As at 08.08.2023) 34313331 10.1002/14651858.CD010783.pub3 PMC8406467

[57] Jessen F (2019) Early detection of Alzheimer‘s disease and approaches for prevention. Bundesgesundheitsbl 62:255–260. https://doi.org/10.1007/s00103-019-02877-2 (As at 08.08.2023) 10.1007/s00103-019-02877-2 30680409

[58] van Dyck CH Swanson CJ Aisen P. (2023) Lecanemab in Early Alzheimer‘s Disease. N Engl J Med 388:9–21. https://doi.org/10.1056/NEJMoa2212948 (As at 08.08.2023) 36449413 10.1056/NEJMoa2212948

[59] Rashad A Rasool A Shaheryar M. (2022) Donanemab for Alzheimer‘s Disease: A Systematic Review of Clinical Trials. Healthcare (Basel) 11. https://doi.org/10.3390/healthcare11010032 (As at 08.08.2023) 10.3390/healthcare11010032 PMC9818878 36611492

[60] Riedel-Heller SG (2018) 11.1 Versorgungssituation Demenzkranker in Deutschland. In: Jessen F (Hrsg) Handbuch Alzheimer-Krankheit: Grundlagen – Diagnostik – Therapie – Versorgung – Prävention. De Gruyter Berlin, Boston, P. 565–590 https://doi.org/10.1515/9783110411003 (As at 08.08.2023)

[61] Hajek A Lehnert T Wegener A. (2018) Long-Term Care Preferences Among Individuals of Advanced Age in Germany: Results of a Population-Based Study. Gesundheitswesen 80:685–692. https://doi.org/10.1055/s-0042-124663 (As at 08.08.2023) 28268234 10.1055/s-0042-124663

[62] Georges D (2022) The effect of informal caregiving on physical health among non-migrants and Ethnic German Immigrants in Germany: a cohort analysis based on the GSOEP 2000 – 2018. BMC Public Health 22:121. https://doi.org/10.1186/s12889-022-12550-0 (As at 08.08.2023) 35042500 10.1186/s12889-022-12550-0 PMC8764847

[63] Brodaty H Donkin M (2009) Family caregivers of people with dementia. Dialogues Clin Neurosci 11:217–228. https://doi.org/10.31887/DCNS.2009.11.2/hbrodaty (As at 08.08.2023) 19585957 10.31887/DCNS.2009.11.2/hbrodaty PMC3181916

[64] Grässel E (1998) Home care of demented and non-demented patients. II: Health and burden of caregivers. Z Gerontol Geriatr 31:57–62. https://doi.org/10.1007/s003910050019 (As at 08.08.2023) 9553225 10.1007/s003910050019

[65] Leicht H König HH (2012) Costs of illness in dementia from a societal perspective. An overview. Bundesgesundheitsbl 55:677–684. https://doi.org/10.1007/s00103-012-1472-9 (As at 08.08.2023) 10.1007/s00103-012-1472-9 22526856

[66] Schwinger A Klauber J Tsiasioti C (2020) Pflegepersonal heute und morgen. In: Jacobs K Kuhlmey A Greß S. (Hrsg) Pflege-Report 2019. Springer, Berlin, Heidelberg, P. 3–21

[67] Mot E Geerts J Willeme P (2012) Long-term care use and supply in Europe: projection models and results for Germany, the Netherlands, Spain and Poland. ENEPRI Bericht Nr. 116. https://ssrn.com/abstract=2060145 (As at 23.08.2023)

[68] Bundesministerium für Familie, Senioren Frauen und Jugend, Bundesministerium für Gesundheit (2020) Nationale Demenzstrategie, Berlin. https://www.bmfsfj.de/bmfsfj/service/publikationen/nationale-demenzstrategie-165218 (As at 22.08.2023)

[69] Ziegert N Hofbauer L Rodriguez FS (2021) Innovative Angebote für Menschen mit Demenz in Deutschland. Nervenheilkunde 40:870–883. https://doi.org/10.1055/a-1529-3414 (As at 08.08.2023)

[70] Spanier H Krah K Nicolas K. (2021) Welche technische Unterstützung gibt es für Menschen mit Demenz? DNP 22:28–34. https://doi.org/10.1007/s15202-020-4610-5 (As at 08.08.2023)

[71] Schulz-Nieswandt F Köstler U Langenhorst F. (2012) Neue Wohnformen im Alter. Wohngemeinschaften und Mehrgenerationenhäuser. Kohlhammer Verlag, Stuttgart

[72] Wolf-Ostermann K Meyer S Schmidt A. (2017) Nutzer und Nutzerinnen regionaler Demenznetzwerke in Deutschland: Erste Ergebnisse der Evaluationsstudie DemNet-D. Z Gerontol Geriatr 50:21–27. https://doi.org/10.1007/s00391-015-1006-9 (As at 08.08.2023) 26779703 10.1007/s00391-015-1006-9

[73] Köhler L Meinke-Franze C Hein J. (2014) Does an interdisciplinary network improve dementia care? Results from the IDemUck-study. Curr Alzheimer Res 11:538–548. https://doi.org/10.2174/1567205011666140618100727 (As at 08.08.2023) 24938504 10.2174/1567205011666140618100727 PMC4150489

[74] Jacobs AH Emmert K Baron R. (2020) Neurogeriatrics – a vision for improved care and research for geriatric patients with predominating neurological disabilities. Z Gerontol Geriatr 53:340–346. https://doi.org/10.1007/s00391-020-01734-1 (As at 08.08.2023) 32430766 10.1007/s00391-020-01734-1 PMC7311516

[75] Schlemm S Kuhlmey A (2005) Demenz – medizinische und pflegerische Versorgung. Gesundheitsökonomie und Qualitätsmanagement 10:238–244. https://doi.org/10.1055/s-2005-858512 (As at 08.08.2023)

[76] Jessen F (2011) Diagnostik der Demenz. Psychiatrie und Psychotherapie up2date 5:9–17. https://doi.org/10.1055/s-0030-1265948 (As at 08.08.2023)

[77] Rapp I Klein T (2020) Lebensstil und Gesundheit. In: Kriwy P, Jungbauer-Gans M (Hrsg) Handbuch Gesundheitssoziologie. Springer VS, Wiesbaden, P. 193–211

[78] World Health Organization (2022) A blueprint for dementia research, Geneva. https://www.who.int/publications/i/item/9789240058248 (As at 23.08.2023)

[79] Epping J Geyer S Eberhard S. (2021) Completely Different or Quite Similar? The Sociodemographic Structure of the AOK Lower Saxony in Comparison to the General and Working Population in Lower Saxony and the Federal Republic of Germany. Gesundheitswesen 83:S77–S86. https://doi.org/10.1055/a-1553-3565 (As at 08.08.2023) 34695865 10.1055/a-1553-3565

